# Supplementary figures and images for: Global analysis of contact-dependent human-to-mouse intercellular mRNA and lncRNA transfer in cell culture
Source: eLife. 2023 May 30;12:e83584. doi: 10.7554/eLife.83584 (PMC10259485; doi:10.7554/eLife.83584)

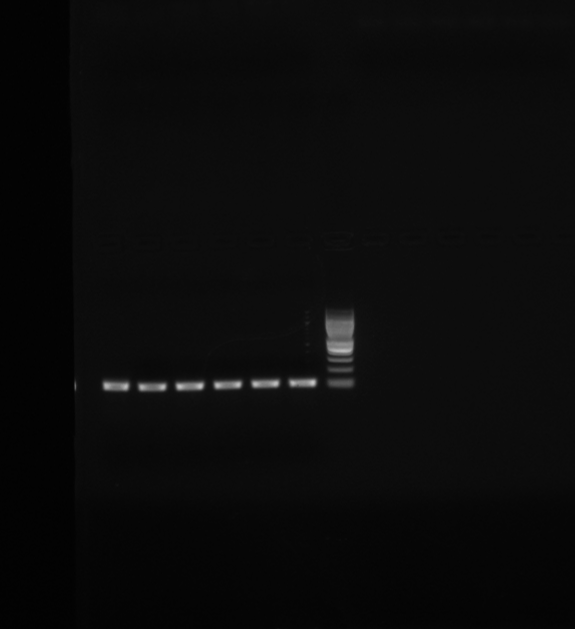

Supplement: Figure 1—source data 1. — The source data contains the original images of the hActb RT-PCR and 18 S RT-PCR gels, and an annotated figure indicating the lane names and the bands corresponding to the specific genes. * indicated non-specific band. [file elife-83584-fig1-data1.zip › Figure 1 - Source File 2 - 18S.tif]

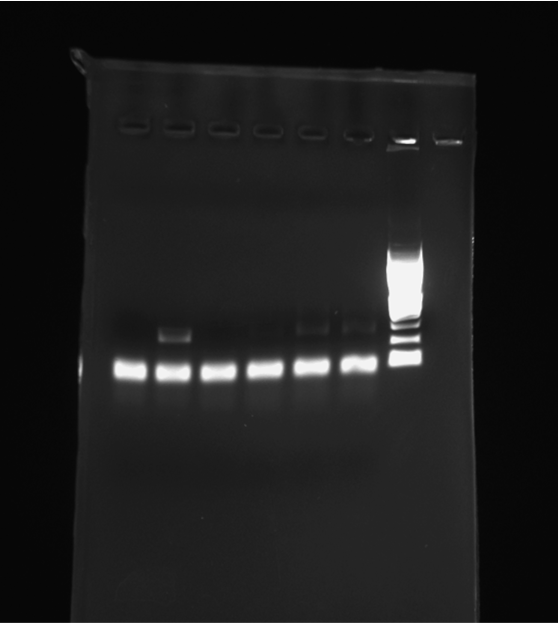

Supplement: Figure 1—source data 1. — The source data contains the original images of the hActb RT-PCR and 18 S RT-PCR gels, and an annotated figure indicating the lane names and the bands corresponding to the specific genes. * indicated non-specific band. [file elife-83584-fig1-data1.zip › Figure 1 - Source File 2 - ActB.tif]

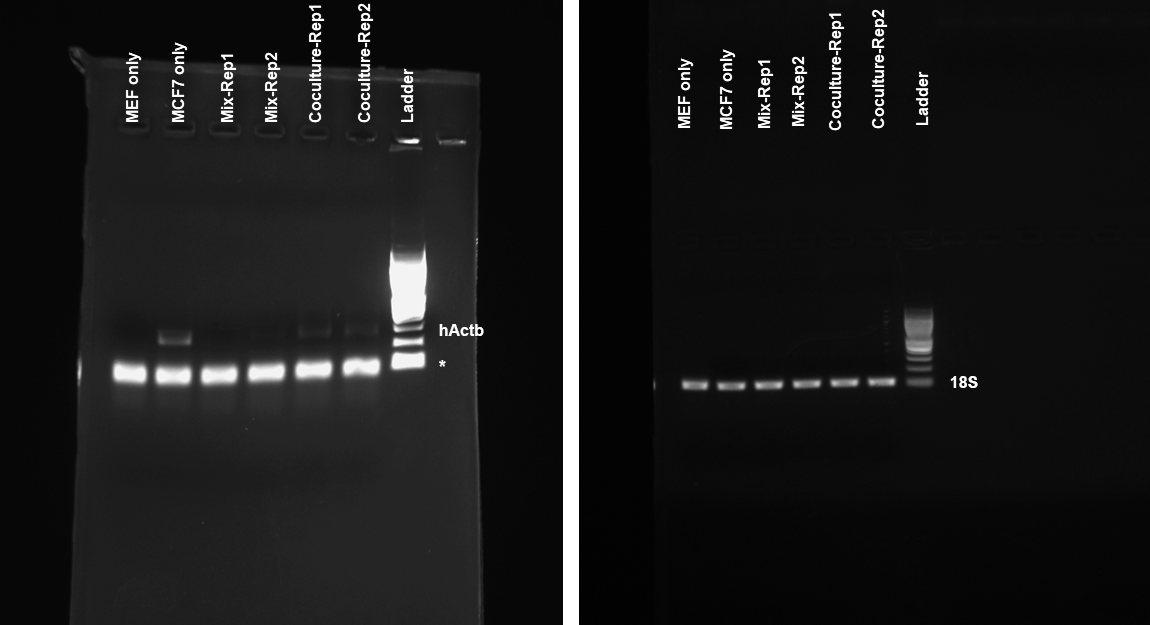

Supplement: Figure 1—source data 1. — The source data contains the original images of the hActb RT-PCR and 18 S RT-PCR gels, and an annotated figure indicating the lane names and the bands corresponding to the specific genes. * indicated non-specific band. [file elife-83584-fig1-data1.zip › Figure 1-Source File 1.tif]
